# Unsalaried health workers in Sierra Leone: a scoping review of the literature to establish their impact on healthcare delivery

**DOI:** 10.1186/s12939-023-02066-3

**Published:** 2023-12-09

**Authors:** Pieternella Pieterse, Federico Saracini

**Affiliations:** School of Nursing, Psychotherapy and Community Health, Dublin City University, Dublin, Ireland

**Keywords:** Unsalaried Health workers, Sierra Leone, Health systems, Healthcare Financing, Access to Healthcare, Universal Health Coverage

## Abstract

**Background:**

The World Health Organisation (WHO) estimates a 10 million health worker shortage by 2030. Despite this shortage, some low-income African countries paradoxically struggle with health worker surpluses. Technically, these health workers are needed to meet the minimum health worker-population ratio, but insufficient job opportunities in the public and private sector leaves available health workers unemployed. This results in emigration and un- or underemployment, as few countries have policies or plans in place to absorb this excess capacity. Sierra Leone, Liberia and Guinea have taken a different approach; health authorities and/or public hospitals ‘recruit’ medical and nursing graduates on an unsalaried basis, promising eventual paid public employment. 50% Sierra Leone’s health workforce is currently unsalaried. This scoping review examines the existing evidence on Sierra Leone’s unsalaried health workers (UHWs) to establish what impact they have on the equitable delivery of care.

**Methods:**

A scoping review was conducted using Joanna Briggs Institute guidance. Medline, PubMed, Scopus, Web of Science were searched to identify relevant literature. Grey literature (reports) and Ministry of Health and Sanitation policy documents were also included.

**Results:**

36 texts, containing UHW related data, met the inclusion criteria. The findings divide into two categories and nine sub-categories: Charging for care and medicines that should be free; Trust and mistrust; Accountability; Informal provision of care, Private practice and lack of regulation. Over-production of health workers; UHW issues within policy and strategy; Lack of personnel data undermines MoHS planning; Health sector finance.

**Conclusion:**

Sierra Leone’s example demonstrates that UHWs undermine equitable access to healthcare, if they resort to employing a range of coping strategies to survive financially, which some do. Their impact is wide ranging and will undermine Sierra Leone’s efforts to achieve Universal Health Coverage if unaddressed. These findings are relevant to other LICs with similar health worker surpluses.

**Supplementary Information:**

The online version contains supplementary material available at 10.1186/s12939-023-02066-3.

## Introduction

The World Health Organisation (WHO) estimates a projected shortfall of 10 million health workers by 2030, mostly in low- and lower-middle income countries [1]. At a global level, the demand for health workers is a top agenda item as countries in the Global North are being criticized for recruiting personnel from Global South countries that face critical shortages [2] and as many struggle to attain their commitments to providing Universal Health Coverage (UHC) by 2030 [1, 3–5]. Beyond the headlines that focus on worldwide shortages of health workers overall, however, a paradoxical oversupply of health workers exists [6]. This ‘surplus’ of health workers, as the WHO calls it, primarily exist in Low-Income Countries (LICs) where the number of medical and nursing graduates who seek employment (the ‘supply’ of the health workforce) exceeds these countries’ ‘demand’ (the total number of public or private sector jobs for health professionals) [7]. A number of African countries is thus faced with a ‘need’ for these health workers, based on the size of the population and WHO guidance on health worker density [8], but many of their trained and qualified health workers are unable to find formal, paid, health sector employment. The health workforce discussed here does not include the many ‘lay, volunteer or community health worker’ cadres, whose efforts, rightly or wrongly, often goes unremunerated, or is compensated through the provision of a small stipend or benefits in kind [9].

The gap between the ‘need’ for health workers (usually calculated based on WHO guidance, and depending on the size of countries’ population), and the ‘demand’ (the actual jobs available) results in many African countries falling short of meeting the WHO recommended 44.5 doctors, nurses and midwives per 10 000 inhabitants ratio, which could enable more countries meeting critical targets such UHC and the Sustainable Development Goals (SDGs) [10]. A shortage of funding for healthcare is often the main reason for countries not meeting minimum health worker/population ratio. Only two countries in Sub-Saharan Africa (SSA) met the spending targets set in Abuja in 2015, allocating 15% of the government budget to health in 2020 [11]. For many SSA countries, the amount of funding that is actually spent on healthcare is further undermined by weak budget execution (many LMICs only spend 85–90% of the funds they budget for health on the sector annually) [12, 13] and government spending on health being captured by a range of corrupt practices [14, 15]. Poor human resources for health (HRH) management means that some countries may have significant numbers of so-called ghost workers on their payroll, who are real or fictitious persons who never carry out the job there are being paid for, but whose salary is collected by the individuals, a relative or the person within the government system who created the ghost worker(s) [16, 17]. Ghost workers can drain a health system’s payroll, while health facilities suffer health worker shortages and ministries of health have little opportunity to recruit new graduates.

The 2016 WHO ‘Health Workforce Strategy 2030’ estimates that the ‘supply’ of qualified health workers will exceed demand in the WHO African Region by about approximately 0.7 million by 2030 [7, 18]. Few countries have developed policies or practices to harness the potential of unemployed health workers and as a result, many trained healthcare professionals either emigrate [19], find themselves unemployed [20] or eking out a living as, for example, drug shop vendors [21, 22]. Several West African countries have adopted a strategy to retain health workers by employing them on an unsalaried basis, with a promise of paid employment further down the line [23, 24]. According to a World Bank report, in 2015/16 the share of health workers who are ‘employed but not on the payroll’ amounted to 39% of the total public sector health workers in Guinea, 44% in Liberia and 48% in Sierra Leone [25]. In these countries, thousands of trained and qualified health workers, perhaps tens of thousands, are technically unemployed but work with little or no official remuneration [26–29]. These unremunerated employees are thought to play a pivotal role in providing care in rural and underserved locations, yet little is known about them. Despite that fact that unsalaried health workers (UHWs) make up a significant number of the total health workforce in each of these three countries, literature that focuses solely on UHWs is scarce. As a result, it is unknown what the impact of the use of such a large unsalaried cohort of workers is, within a public health service. This scoping review focuses on the impact of UHWs on Sierra Leone’s health system. Sierra Loene has officially acknowledged in government policy and strategy papers that since 2016, almost half of its health workforce consists of unsalaried workers [29].

### Background to Sierra Leone

Sierra Leone is a small West African country with a population of approximately 8 million [30]. The country emerged from a brutal civil war in 2002 and has been stable and peaceful since. Immediately post-war, Sierra Leone was briefly in receipt of the largest per-capita aid donations globally [31]. Despite the significant investment in the post-war re-establishment of the health system, Sierra Leone’s health outcomes languish at the bottom of many league tables. Sierra Leone is among the ten worst performing countries when it comes to neonatal mortality, infant and under-five mortality, stillbirth and child death rates [32–34]. The maternal mortality rate, once the worst in the world, has improved recently with a reduction to 717 per 100,000 live births in 2019 [35] and 443 per 100,000 live births in 2020 [36], however, structural improvements that could bring further reductions have been slow [37]. The country suffered a devastating Ebola Virus Disease (EVD) outbreak between 2014 and 2016, in which at least 4,000 citizens and 221 health workers died [38].

Sierra Leone’s health system has a long history of challenges; starting off worse than many other African states, post-independence [39] and declining before and during the 11-year long civil war from 1991 to 2002, in which many professionals fled - especially its better educated health workforce [40]. It is thought that the few doctors and nurses who stayed during the war resorted to dual or private practice at times and charged for their services to make a living when their public salaries went unpaid for prolonged periods [41]. The post-war period was initially reconstruction-focused, while the health system itself received less attention, which meant care remained out of reach for many patients who could not afford to pay [42]. In 2010, Sierra Leone introduced the Free Healthcare Initiative [FHCI] for children under five, and pregnant and lactating mothers, in a bid to make primary healthcare more equitable and accessible [41]. The target community for FHCI was expanded in 2016 to include additional ‘vulnerable’ groups such as Ebola survivors [43].

The introduction of the FHCI marked significant changes. The FCHI-linked payroll cleansing in 2010 removed many ghost workers, and the subsequent payroll expansion allowed for the recruitment of new health workers. Many trained healthcare staff who had previously worked as so-called ‘volunteers’ were put on the payroll, significantly reducing Sierra Leone’s reliance on unsalaried staff, who were known to be charging for their services [44]. The promise of free healthcare attracted patients to health facilities in much greater numbers than before. While the initial spike in healthcare uptake did not last, the demand for healthcare provided by health facilities and hospitals (as opposed to traditional healers and other informal care providers) has remained at a much higher level since 2010 [45, 46].

Despite the greatly improved HRH system put in place in 2010, the practice of recruiting new ‘volunteers’ in the public health system on an unsalaried basis did not stop. In 2016, a report titled *Sierra Leone Human Resources for Health, Country Profile*, published by the Ministry of Health and Sanitation (MoHS), revealed that only half of its health workforce is on the payroll and out of the 9,120 unsalaried health workers identified in the payroll audit, approximately 40% were health professionals providing patient services, primarily in the lower-skilled cadres [29].

Sierra Leone’s health systems currently consists of over 1,400 facilities, including several specialist and referral hospitals, district hospitals, and primary healthcare is provided through a network of Peripheral Health Units (PHUs) [47]. The private sector provision of healthcare is relatively small and private facilities are primarily found in the capital Freetown and several other cities [44]. Sierra Leone’s health systems, including its human resource management, is highly centralised, due to the stalling of the implementation of the 2004 decentralisation legislation [48, 49]. District Health Management Teams (DHMTs) cannot hire staff, decide where to deploy health workers, or dismiss them even if they misconduct themselves or are absent [50, 51] which leaves them lacking “power, resources and institutional incentives to enforce formal rules” [52]. Despite significant post-Ebola health systems strengthening support from the donor and NGO community, Sierra Leone’s health facilities remain under-resourced and underperforming. Official user fee exemptions exist, but charging for healthcare has remained a problem, and those exempted often still pay [53, 54]. A study carried out in 2016 found that “as many as 96% of households responded they paid” for supposedly free services [50], suggesting that charging for care is widespread and seemingly practiced by salaried and unsalaried workers alike. If Sierra Leone were to introduce greater monitoring and oversight of its health workers to curtail informal charging, the question remains; how can unsalaried workers cope without pay for prolonged periods of time? Health service provision in remote and rural would likely be much reduced without the contribution of UHWs, however, their coping mechanism might also be one of the barriers to the implementation of Sierra Leone’s free healthcare initiative [54]. This study assesses the influence that a significant reliance on UHWs has on the delivery of healthcare in Sierra Leone by providing an overview of all available evidence in the literature that demonstrate positive and negative impacts.

## Methodology

A scoping review methodology was selected because this topic requires assessing and understanding “…the extent of the knowledge in an emerging field or to identify, map, report, or discuss the characteristics or concepts in that field” [55]. The first methodological guidance on conducting scoping reviews was published in 2005 by Arksey and O’Malley [56], which was subsequently refined and further structured by Levac et al. [57]. In the past decade, the JBI International Scientific Committee has been instrumental in tracking developments in the use of the scoping review approach and providing updated guidance as new developments emerge [55, 58]. This scoping review follows JBI’s latest guidance [59] and uses the 2018 Preferred Reporting Items for Systematic Reviews extension for Scoping Reviews [PRISMA-ScR] checklist [60] to transparently demonstrate adherence to all important steps of the scoping review process [additional file 1].

This scoping review was registered via the Open Science Framework: 10.17605/OSF.IO/X2G6P and is guided by the following research question:

*How does having a significant number of unsalaried health workers affect the quality of healthcare delivery and health outcomes, in Sierra Leone*? *What evidence is there to demonstrate this impact?*

The research question reflects the title of this study, “UHWs in Sierra Leone: a scoping review of the literature to establish their impact on healthcare delivery” and contains references to the scoping review’s three key elements [55], its *Population*: qualified health workers [with a minimum Maternal and Child Health Aide (MCHA) qualification], who are employed on an unsalaried basis in a Sierra Leonean public sector health facility; the *Concept*: the range of impacts that the unsalaried status of many health workers has on their behaviour, and their ability to carry out their work; and the *Context*: Sierra Leone’s public health system, since the end of the civil war, from the time its current ‘health system’ was established.

Despite the omnipresence of UHWs in Sierra Leone, no studies have explicitly focused on them, or examined their impact. This scoping review is therefore not an analysis of studies that are explicitly about UHWs, but instead a review of all ‘UHW related references’ contained *within* studies, peer-reviewed articles, reports and policy documents about Sierra Leone. While not many scoping reviews take this shape, the scoping review method was nevertheless found to be particularly suitable for this task, as it allows authors to “incorporate various types of literature that are not limited specifically to research studies” [57].

For this review, the Medline, PubMed, Scopus, Web of Science databases were searched for the key terms, and appropriate MeSH terms were used (Table 1).

**Table 1:** Sample search strategy

Searches	Search terms
Search 1:	“Sierra Leone”
Search 2:	“healthcare staff” OR “health worker” “healthcare worker” OR “human resources for health” OR nurse OR doctor OR Pharmacist OR “MCH Aide” OR “State-Enrolled Community Health Nurse”
Search 3:	Search 1 AND search 2

The identified peer reviewed text references (n = 587) were then entered into Covidence software, which was used to check all texts for suitability against the inclusion and exclusion criteria (Table 2).

**Table 2:** Inclusion/exclusion criteria

**Inclusion criteria**	
1	A document contains [a] reference[s] to qualified (minimum MCH Aide cadre) unsalaried, or volunteer health workers, or health workers who are not on payroll
2	In the English language
**Exclusion criteria**	
1	Any study not focused on Sierra Leone
2	Any study published before 2002 (which is therefore not focused on Sierra Leone’s ‘post-war health system’)
3	Any documents in which the term ‘volunteer’ refers to Community Health Workers, or other type of lay healthcare provider, or international volunteer

Initial checks focused on title and abstract selection of suitable texts, PP screened all, a second reviewer, FS, checked 10% of the documents to verify if the inclusion/exclusion criteria were correctly applied. PP and FS jointly resolved disagreements during a face-to-face meeting. A full text screening of 90 texts was carried out. To ensure no mentions of the key words were overlooked, NVivo12™ software was used to check all 90 texts for any occurrences of the words *unsalaried*, *volunteer* and *payroll*. Any texts containing these words were further checked to verify whether these words related to the type of UHWs (not-on-payroll) on which this scoping review focuses.

The total number of peer reviewed texts identified during the document search was 23. Several relevant MoHS policy and strategy documents were included in this review, n = 6, as were reports by reputable ‘grey literature’ sources such as UN agencies, think tanks and advocacy groups, n = 5. Two additional peer reviewed articles were added to the body of literature, because they contain references to UHWs but were not captured in the literature search.

**Fig. 1:**
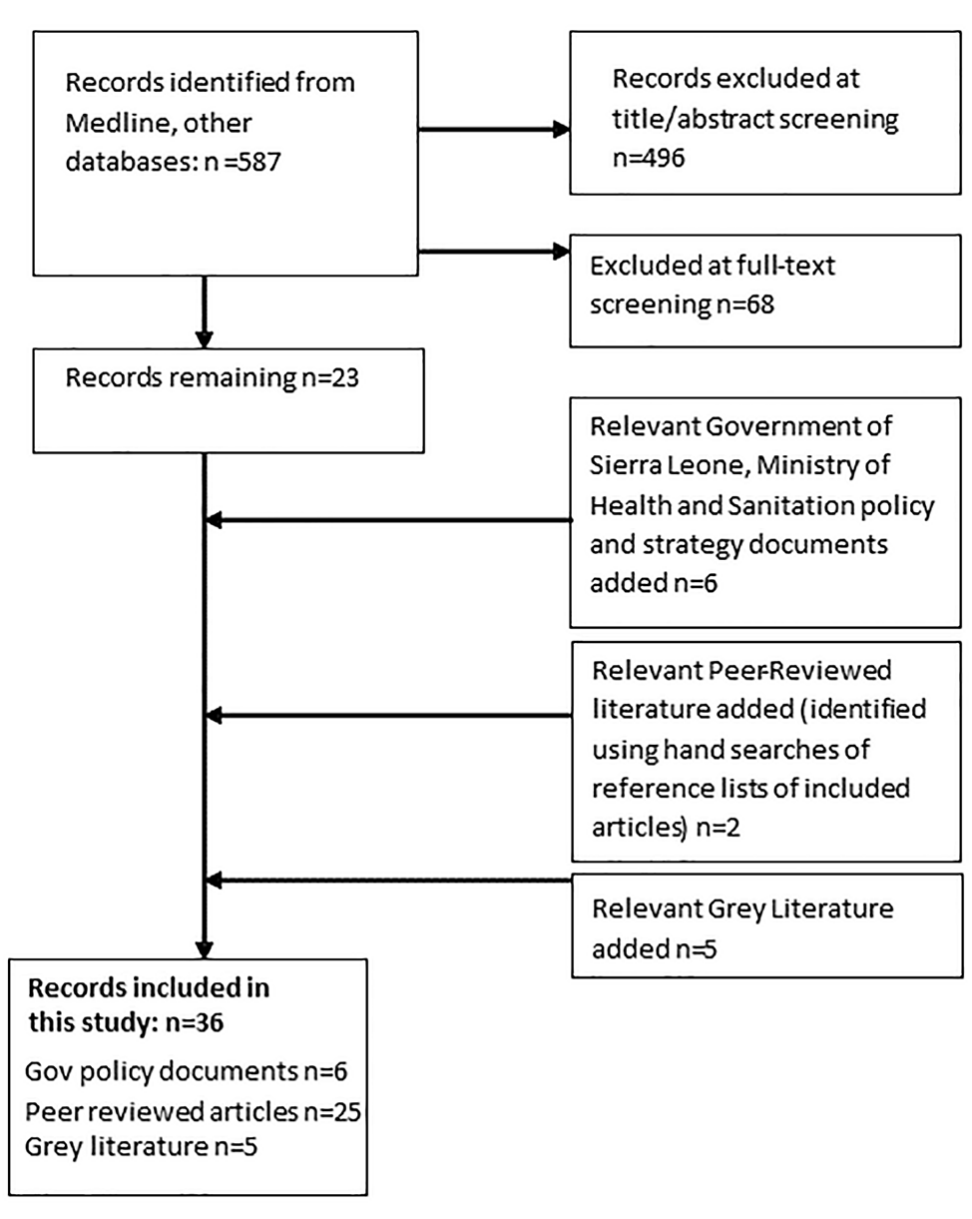
The flow diagram for the literature search

Figure 1 contains the Flow Diagram of the literature search for this review.

## Findings

A total of 36 texts were included in this review, and UHWs were referenced in all of these. The list of all included articles can be found in additional file 2. The peer reviewed and grey literature text (n = 30 in total) focus primarily (90%, n = 27) on the ‘post-FHCI period’. A total of 13 text focused on a PHU setting, seven on a hospital setting, seven on both PHU and hospital and, one focused on a COVID-19 treatment centre. Two papers focused on policy; this classification excludes the MoHS policy documents. A disproportionate number of hospital-based studies (n = 4) focused on Connaught Hospital, Sierra Leone’s main tertiary referral hospital, reflecting the hospital’s continued engagement with several Global North university or NGO-led hospital-based capacity strengthening partnerships.

### UHW references

To understand the types of references to UHWs in the included texts, notable excerpts were extracted, which led to the identification of categories of evidence, see Table 3.

**Table 3:** Categories of evidence in the literature regarding UHWs

Categories of evidence	Sub-categories
*A-Unintended consequences in relation to UHWs*	1. Charging for care that should be free 2. Charging for ‘free’ medicines/record cards 3. Mistrust as a result of informal charging 4. Accountability 5. Informal provision of care, private practice and lack of regulation
*B-Impact on (and of) national policies and strategies regarding UHWs*	6. Over-production of health workers 7. UHW issue highlighted and/or included in policy and strategy 8. Lack of HHR data undermines MoHS planning 9. Healthcare financing and UHWs: Performance Based Finance

The frequency with which each of the categories of evidence was found in the included texts, and details about the context in which UHWs were mentioned in each text, can be seen in Table 4.

**Table 4:** Details related to UHWs references in the texts included in this scoping review

	Author, year, title	Type of document	Significance of reference to unsalaried health workers [UHWs]:	A: Unintended consequences in relation to unsalaried health workers					B-Impact on (and of) national policies and strategies regarding unsalaried health workers			
				1. Informal charging for healthcare provided to FHCI target groups	2. Charging for medicines or vaccines [+ vaccine cards]	3. UHWs undermining trust in public healthcare provision	4. Lack of accountability of UHWs	5. Informal provision unregulated care/private practice	6. Over-production of health workers	7. UHW issue highlighted and/or included in policy and strategy	8. Lack of HHR data on UHW undermines MoHS planning	9. Healthcare financing and UHWs: Performance Based Finance
1	GoSL, MoHS 2016, Human Resources for Health Summit, 2–3 June 2016 Freetown, Sierra Leone	Gov policy/Min of Health & Sanitation report	Substantial references of UHW in detail							"Removing [ghost] health workers from the payroll should result in an annual saving of approx. $2 million (10% of the MOHS wage bill), freeing up fiscal space to absorb volunteers into the health workforce".	…some transfer decisions can be made at the district level and “informal recruitment” of unemployed health workers volunteering takes place at the facility level without prior knowledge at the national level. Workforce data management was also described as a priority challenge, with iHRIS currently being introduced and institutionalized to redress the situation.	
2	GoSL, MoHS 2016, Human Resources for Health Country Profile Sierra Leone	Gov policy/Min of Health & Sanitation report	Substantial references of UHW in detail						“…the overproduction of SECHNs has led to a significant rate of unemployment for these nurses: roughly 2,000 unsalaried, licensed SECHNs were found to be practicing in government facilities… The coexistence of SRN shortages with SECHN unemployment highlights the need for better coordination of pre-service training plans with MoHS health worker production needs.”	A MoHS payroll audit suggests as many as 9,120 UHWs are active in Sierra Leone’s government health facilities, out of 19,030 public sector health workers. Of the 9,120 UHWs, 3,690 (40%) serve as health professional providing patient services, primarily in the lower-skilled cadres.		
3	GoSL, MoHS 2016, Human Resources for Health Strategy 2017–2021	Gov policy/Min of Health & Sanitation report	Policy proposal re UHWs in detail				“Roughly half of health workers active in government facilities are unsalaried and, hence, not part of the formal health workforce – making it difficult to supervise and regulate these workers.”	In ref to UHW: “The HRH strategy should thus include a clear policy statement to address these unemployed, and thus unregulated, health workers”… ”[HW] production often exceeds the government’s absorption capacity – particularly for lower cadre health workers. This has resulted in over 3,600 unsalaried clinical health workers providing patient services in government facilities despite not being part of the formal MoHS workforce.”	… no progress on “establishment of a nationally coordinated pre-service training plan since 2011. As a result, production often exceeds the government’s absorption capacity – particularly for lower cadre health workers” … ”professional [health training institutions] regulatory bodies remain under-resourced, meaning some [medical training] programmes continue to operate without accreditation, and the licensing process is challenging to enforce and monitor.”	“Cost estimate: USD 6 million over 5 years on salaries resulting from the absorption of unsalaried health workers in critical cadres… Develop and implement a plan for absorption of critical unsalaried workers, i.e. workers trained as SRNs, midwives, or CHOs, and a policy on remaining unsalaried workers – including relevant management and monitoring procedures for enforcement”	[2016 Payroll verification] “resulted in a cleaned payroll dataset of all MoHS staff, a comprehensive database of the existing health workforce – including the unsalaried workforce – and updated guidelines and standards of practice to streamline and maintain payroll”	
4	GoSL, MoHS 2017, Sierra Leone Service Availability and Readiness Assessment [SARA]	Gov policy/Min of Health & Sanitation report	Substantial references of UHW in detail							"There was a total of 323 general and specialist doctors in the country. It is notable that 35% were not formally employed. They offered their services either on a part-time basis, paid by the facility management or a nongovernmental agency, or on a volunteer basis."		
5	GoSL, MoHS 2021, National Health Policy 2021–2025	Gov policy/Min of Health & Sanitation report	Short reference	“Despite the introduction of the FHCI in 2010 to reduce the financial burden of accessing care by the vulnerable groups, the cost of care is still considered to be out of the financial reach of the majority of the population, posing financial risk to them. It is estimated that households out of pocket expenditures contribute 61% of total health expenditure in Sierra Leone (NHA 2018 study) one of the highest in sub-Saharan Africa.”						“The Directorate of Human Resources for Health (DHRH) developed a national HRH Policy and an HRH Strategy to address the challenges affecting healthcare workers in Sierra Leone from 2017–2021. The HRH Strategy 2017–2021 provides a framework to guide investments and activities to achieve the vision, goal and objectives set forth in the HRH Policy 2017–2021.”		
6	GoSL, MoHS 2021, National Health Sector Strategic Plan 2021–2025	Gov policy/Min of Health & Sanitation report	Policy proposal lacks detail re UHWs	Reference to Out-of-Pocket Expenditure.					“An annual multi-stakeholder HRH forum for medical training institutions and other stakeholders may be held to assess intake numbers and priority areas, to match the sector needs. This may include both pre-service and in-service training to upgrade competencies of certain health workers to those most needed, such as more midwives, registered nurses, medical doctors, and biomedical scientists/technicians, among others, focusing on quantity and quality.”	“It was estimated that there are around 20,000 health workers in Sierra Leone who work in a variety of cadres; approximately 50% are volunteers and not on the government payroll” [2016 data].	“Health workforce performance management and accountability may be strengthened, based on a comprehensive review of staffing norms, making use as needed of the Workload Indicators of Staffing Needs (WISN). A facility-by-facility health workforce gap analysis may be conducted annually to inform HRH recruitment and deployment.”	
7	Bakker et al. 2021, Barriers to increase surgical productivity in Sierra Leone: a qualitative study	Peer reviewed article	Refs to UHW waiting for inclusion on payroll and upskilled surgical providers whose salaries not adjusted upward to reflect new job/responsibilities	Financial barriers observed for surgery, both at patients demand side and hospitals provision side. + Gen observation that FHCI care is not always free	FHCI patients pose financial pressure on facilities; required supplies from government are insufficient. Costs passed on to patients, decreasing access to surgery		“Informal system of care providers exists within and outside of public health facilities.”	“Informal/unlicenced ‘mushroom’ clinics in community delay necessary care.” “Financial incentives to not record surgical activities in hospital logbooks”: under-registration of surgeries				
8	Bertone and Witter 2015, An exploration of the political economy dynamics shaping health worker incentives in three districts in Sierra Leone	Peer reviewed article	HRH focus, ref to 15% of HW not on payroll during 2013 research		Access to drugs = powerful financial incentive for health workers if there is room for misappropriation and informal sale							
9	Bertone and Lagarde 2016, Sources, determinants and utilization of health workers’ revenues: evidence from Sierra Leone	Peer reviewed article	In-depth log-book data from 266 HWs on their sources of income incl. informal. …odds of receiving salary are 11 x larger for in-charges [i.e. the facility managers] compared to staff workers, less for MCHPs than CHCs	In-charges or CHOs more likely to receive gifts from patients: “the fact that 74% of workers declared to be receiving [gifts] and their estimate at 3–5% of total revenues seems to be reliable with reference to another aspect of the relation between HWs and patients/communities”	“…odds of earning income from selling drugs are almost 20 times larger for CHOs compared to CHAs/nurses and MCH Aides”			Among income sources for HW is private practice; more common among higher cadres such as doctors, CHOs				
10	Bertone, Lagarde and Witter 2016, Performance-based financing in the context of the complex remuneration of health workers: findings from a mixed-method study in rural Sierra Leone	Peer reviewed article	Short ref to PBF payments being perceived as salary/payment for unsalaried HWs									"In some cases, health workers posted immediately after training were given individual [PBF] bonuses despite not being eligible for it as not working in facility when the bonus was accrued. This practice was justified by the fact that they were not yet on payroll and would have little alternative financial means to support themselves."
11	Brooks and Herrick 2019, Bringing relational comparison into development studies: Global health volunteers’ experiences of Sierra Leone.	Peer reviewed article	Substantial refs to UHW in context of international medical volunteers experiencing tertiary hospital in Freetown	“An informal system of nursing care and Medication distribution had developed. Unsalaried volunteer nurses drew an income from patients”	“Many Sierra Leonean nurses went unpaid and sold medicines and supplies ‘from their handbags’.”		Shortcomings re staff, too many UHWs, lack of supplies, electricity, water- unrealistic to expect ‘teaching hospital quality services’					
12	Dorwie and Pacquiao 2014, Practices of Traditional Birth Attendants in Sierra Leone and Perceptions by Mothers and Health Professionals Familiar with Their Care	Peer reviewed article	Refs to TBAs accepting any kind of payment whereas health practitioners require upfront fees [pregnant women entitled to FCHI free care]	“TBAs [Traditional Birth Attendants] are affordable care providers who accept any form of payment (cash, goods, or services). Unlike HPs who require *standardized, upfront fees*, TBAs *give services first*, and families *can pay later based on what they can afford*.”		[UHW lacking trust in health system themselves] “One physician commented that we have to look at the underlying problem. People access care only when they are in dire need; the doctors are greedy and make it difficult for the poor and the needy. The country is full of corruption, one works and expects to get paid at the end of the month, but this is not the case.”	This paper focuses on TBAs and highlights some women’s preference to receive TBA care, as it is perceived to be cheaper and they offer flexible payment options- this demonstrates that pregnant women may opt seek potentially unsafe TBA care if healthcare workers are found to charge for care that should be free.	“All groups of participants disdained TBAs *motivated by money, placing the mother and baby* in jeopardy. Each group of participants described several incidents when mothers suffered complications (e.g., *bleeding, sepsis, rupture or prolapsed of the uterus, fetal and maternal death*) because of *failure of TBAs to make timely referral to HPs*. HPs gave examples of unsafe TBA practices as *forcing labor, unsanitary conditions, condoning superstitious and unhealthy cultural practices, use of toxic chemicals (kerosene), dangerous remedies (papaya leaves, animal dung), witchcraft.”*				
13	Elston et al. 2020, Maternal health after Ebola: unmet needs and barriers to healthcare in rural Sierra Leone	Peer reviewed article	Barriers; cost* + inaccessibility, “delayed/prevented 90% rural, 59% urban pregnant women from receiving healthcare [n = 608].” *Cost = Lack of money for either paying for a consultation with a healthcare worker (HCW).	Refs to pregnant women having to buy medicines from HWs. Also: Ref to ‘informal skilled care being available to urban women, in form off ‘off-duty HWs’’.	Additional maternal care seeking barrier: concerns about being treated disrespectfully by HCWs. Also: additional barriers including lack of medications and HCW absences.	“Women expressed mistrust of HCWs primarily due to payments for ‘free’ healthcare. Despite preference for biomedical care, 48% rural and 31% urban women gave birth outside of a health facility.” Also: HCWs described lack of pay, poor conditions precluding provision of quality care.		“Participants explained a preference for delivering at home with the assistance of a local off-duty nurse, only seeking hospital care when complications were beyond their skills.”				
14	Enria et al. 2021, Bringing the social into vaccination research: Community-led ethnography and trust building in immunization programs in Sierra Leone	Peer reviewed article	Several mentions of UHWs and coping strategies which emerge as contributing to lack of trust in gov health facilities [during Ebola vaccine trial research]	Researchers, who are CHWs uncover “structural issues that underpin mistrust in the health sector”. CHWs recommend the provision of stipends for volunteer health workers [as full time UHW receive less than part-time, semi-trained CHWs]	“…frequent drug stockouts which led to health staff having to ask patients to buy their own, or the fact that nurses were volunteers and at times had to supplement their income by ‘selling’ vaccination cards.”	Respectful, two-way dialogue as step towards restoring trust: both community + HWs “concerns, anxieties and limitations discussed”, e.g. “fact that health workers are often not paid was useful for understanding the challenges to delivering [health] services”						
15	Frankfurther 2019, Conjuring Biosecurity in the Post-Ebola Kissi Triangle: The Magic of Paperwork in a Frontier Clinic	Peer reviewed article	Focus on one remote clinician, expected to “given great autonomy to create auxiliary staffing systems” and “define informal payment schemes”		“Clinicians like Tamba are left to *make do* by crafting their own informal payment plans and contracting with independent pharmaceutical suppliers to keep clinics stocked”							
16	Herrick and Brooks 2018, The Binds of Global Health Partnership: Working out Working Together in Sierra Leone	Peer reviewed article	Brief mention of UHW	“Many of Connaught’s nurses worked as unpaid volunteers often making their living by charging patients informal fees for goods and services [in addition to the formal fees of the hospital]”								
17	Jalloh et al. 2022, Association of community engagement with vaccination confidence and uptake: A cross-sectional survey in Sierra Leone	Peer reviewed article	Brief mention of UHW		Research suggests caregivers expect to pay for their child’s vaccines despite FHCI. Research connects charging for care to UHWs							
18	McPake et al. 2013, Removing financial barriers to access reproductive, maternal and newborn health services: the challenges and policy implications for human resources for health.	Peer reviewed article	Brief mention of UHWs, pointing out that in 2012, few UHWs left, probably due to lack of user fee income									
19	Miller et al. 2018, Community health workers during the Ebola outbreak in Guinea, Liberia, and Sierra Leone	Peer reviewed article	Brief mention in CHW context. Re debating option of paying stipends through gov payroll -point made that even those on payroll don’t all receive salary									
20	Navarayan et al. 2018,“If I had known, I would have applied”: poor communication, job dissatisfaction, and attrition of rural health workers in Sierra Leone.	Peer reviewed article	Rural HW focus, lack of salary emphasised, lack of info about procedures, incl ‘how to get on to the payroll’								Rural HW attrition: 63% of SL’s population is rural, only 33% of HWs work at rural PHU; study emphasises rural disadvantage re HR policies, incl. payroll related for UHWs	
21	Nyhus and Kamara 2017, Quality improvement in emergency service delivery: Assessment of knowledge and skills amongst emergency nurses at Connaught Hospital, Sierra Leone	Peer reviewed article	Emergency nursing assessment in tertiary hospital; nurses note low/no salary, bureaucratic challenges re career opportunities	“Unofficial out-of-pocket payments and a high percentage of the nurses not receiving salary [71%] must be addressed”			Challenges re implementing triage protocol at referral hospital A&E, due to “out-of-pocket payment considerations” from other hospital wards					
22	Oyerinde et al. 2011, The status of maternal and newborn care services in Sierra Leone 8 years after ceasefire	Peer reviewed article	Study notes “unemployed trained health workers [“volunteers”] in public health facilities. They were paid for services by private arrangements with patients and their relatives.”	Despite “national policy states that MCH services are free” pregnant women and sick children paid fees. Chargers “varied widely between facilities and were so unpredictable or arbitrary that patients and their families could not anticipate their OOP expenditure”								
23	Pieterse and Lodge 2015, When free healthcare is not free. Corruption and mistrust in Sierra Leone’s primary healthcare system immediately prior to the Ebola outbreak	Peer reviewed article	Study notes encounters w UHWs, contains UHW interviews, interviews + FGD w FHCI target group healthcare users, who report charging for care	Study notes “charging for free care” allegations during 24 out of 35 FDG	Study notes “charging for free care” allegations during 24 out of 35 FDG; including charges for medicine, vaccine or ante-natal care cards	High levels of mistrust of HWs noted among pregnant women, mothers w < 5 children [FHCI target groups], aware that supposed free care is not being delivered. Mistrust in HWs were thought to have led to evasive actions by some EVD patients and patients’ families, whereby treatment was delayed or not sought and the spread of EVD exacerbated	Study specifically examined ‘accountability promoting interventions’ most of which failed to reduce informal charging, some HW behaviour did improve					
24	Squire et al. 2017, The Ebola outbreak and staffing in public health facilities in rural Sierra Leone: who is left to do the job?	Peer reviewed article	Study on staff shortages in Kailahun District: of 805 recommended medical staff [minimum requirement in 82 PHUs], deficits of 539 [67%] pre-Ebola, 528 [65%] during the Ebola outbreak and 501 [62%] post-Ebola existed								“the issue of non-registered volunteer staff is of concern. If they are not captured by information systems, such staff may … be left out when facility requirements for personal protective equipment are being considered. Volunteers will thus be more susceptible to both acquiring and transmitting infectious diseases to co-workers, patients and the community at large.”	
25	Squire et al. 2020, Staffing in public health facilities after the Ebola outbreak in rural Sierra Leone: How much has changed?	Peer reviewed article	Short study on staff shortages in Kailahun district, 3 years post-Ebola; noting that “nothing has changed”						“…ensure that at the subnational level there are sufficient numbers of candidates from the cadres… These steps should be preceded by a national census of HCWs to identify trained but currently unemployed individuals who could be absorbed into the public services”		“due to budgetary limitations on paying salaries, many health care workers serve as volunteers in health facilities and are not on a regular payroll”	“HCWs not on the payroll do not get paid a salary. Other incentives, such as performance-based financing and stipends for training and national campaigns, may help. These incentives, however, do not constitute a living wage.”
26	Tengbe et al. 2023, Psychosocial impact of COVID-19 pandemic on front-line healthcare workers in Sierra Leone: an explorative qualitative study										[improvements need to successfully respond to future epidemics] “Improving conditions of service, availability of essential medications and supplies, the recruitment of volunteer HCWs not on the government payroll”	
27	Treacy, Bolkan, Sagbakken 2018, Distance, accessibility and costs. Decision making during childbirth in rural Sierra Leone: A qualitative study	Peer reviewed article	Vague ref to charging for care, couched as ‘appreciation’ for HW who are paid *late and if at all, very little’*	Cost as barrier to care seeking during childbirth								
28	Vernooij, Koker, Street 2022, Responsibility, repair and care in Sierra Leone’s health system	Peer reviewed article	Ethnographic study illustrating how to “make the [hospital] system work, for the sake of the patients”	“…scholars have written about the informal payments underpinning transactions in the Connaught Hospital, reporting that unsalaried staff [routinely referred to as ‘volunteers’] draw an income from patients by selling medicines and services”	“The breakdown in government supply systems created a gap in service availability which was patched by health workers, who established a quasi-private testing service within the hospital and took on the responsibility of procuring materials. [It happens at] every site of medical testing in the hospital, ranging from the triage [where nurses sold glucose tests] to the laboratory and radiology department.”							
29	Willot et al. 2021, Staff recognition and its importance for surgical service delivery: a qualitative study in Freetown, Sierra Leone	Peer reviewed article	Study to understand barriers to surgical care from a variety of perspectives, to recommend interventions to improve access and quality of care	Patients: “many health workers [are] unapproachable and uncaring, particularly towards poorer patients who are unable or unwilling to pay staff extra in the form of informal payments for their care”		Low morale leading to poor care: “Low morale is exacerbated by perceptions of a lack of recognition of their work by superiors and by the system itself. Our research suggests that nurses do not feel valued by the management of the hospital or by the MoHS.”						
30	Wilson et al. 2022, Challenges and solutions to providing surgery in Sierra Leone hospitals: a qualitative analysis of surgical provider perspectives.	Peer reviewed article	Focus on surgical capacity, which is hampered by heavy reliance on UHWs				High levels of UHW absenteeism: “volunteers have no official compensation structure, they are difficult to hold accountable for the work they perform”					Large reduction in surgical capacity by only conducting surgery when sufficient staff: “One provider, noting that support staff [many UHWs] tended to leave by the afternoon, scheduled major surgical cases in the morning”
31	Witter et al. 2015, The free health care initiative: how has it affected health workers in Sierra Leone?	Peer reviewed article	Predominantly focused on pre-FHCI HRH conditions, highlights UHW “volunteers” who are subsequently added to payroll			Study connects payment for fees and lack up uptake by highlighting impact of fee removal “*people who were afraid in the former days to come to the hospital because maybe they were not having money, they think they will be charged and so on, are coming in hundreds*”					Study pinpoints lack of long-term HRH solutions: “long-standing issues, such as improving and decentralizing the recruitment, deployment and management of HRH still unresolved”	
32	Amnesty International 2009, Out of Reach: The Cost of Maternal Health in Sierra Leone	Research report	Pre-FHCI report exposing barriers to healthcare access, some of it related to UHW and low pay of all HWs	The Anti-Corruption Commission [2008] “recognized that conditions of service are poor and need to be improved but also found that staff at all levels were unilaterally charging revenue and not reporting it”	The report highlights that donated medical supplies are for sale in markets, while health facilities are out of stock, or patients pay for donated medicines and supplies	The lack of HW pay and poor working conditions has led to “widespread corruption and arbitrary charges… [impacting] quality and availability of health care. The fear of costs, and the refusal of some health workers to treat women without payment even in an emergency, leads to deadly delays in the decision to seek or receive care”	“Poor inventory control management, together with poor accountability and mismanagement of drugs and supplies, have been identified as problems”					
33	Bertone and Witter 2013: The development of HRH policy in Sierra Leone, 2002–2012 – report on key informant interviews	Report published by Rebuild Consortium	Refs to UHWs pre-FHCI. Also the 2011 planned Health Service Commission [HSC], which is still not functioning	[Pre FHCI] *money was a barrier for women and children to access the health service delivery*				“HWs may e.g. refuse to work in rural areas as they may not be able earn additional income from private practice or other sources”				
34	Govindaraj R, Herbst CH, editors. Strengthening post-Ebola health systems: from response to resilience in Guinea, Liberia, and Sierra Leone	World Bank report	2018 review; UHW % of total health workforce: 48% in SL, 44% in Liberia and 39% in Guinea.								Report presents HRH statistics warning it uses formal payroll data to calculate density, but cautions that stats exclude informal HRH	
35	Witter et al. 2016, The Sierra Leone Free Health Care Initiative [FHCI]: process and effectiveness review	Report published by Rebuild Consortium	Extensive [300 page] report that includes finance analysis and highlights role of UHWs in health system around 2015	“Despite much probing, it was also impossible to understand how these volunteers survived financially: some did get a share of the PBF and some did not. They all denied charging under the counter, although one facility did state that patients gave fees to volunteers voluntarily”		”	“In some areas visited… the volunteers are reported to outnumber paid staff” … “This causes difficulties for staff management and accountability”	Research among HW showed that non-salary income such as private practice income, PBF, patient gifts, per diems etc.			However, the [2015] situation has deteriorated since [FHCI introduction] “the recruitment drive has not continued and many staff who joined in recent years are not on payroll. Some new facilities have also opened, which stretches staffing more thinly.”	Survey data from 141 PHUs shows that they spent one-third of income [PBF and cost recovery] “on volunteer incentives and community outreach, highlighting the role of non-formal staff at PHU level” “PBF payments were reported to be motivating, when received, although many reported a long delay since they had last received any PBF. PBF is particularly important to motivate the unsalaried staff”
36	Wurie et al. 2014, Staffing the public health sector in Sierra Leone, 2005-11: findings from routine data analysis’	Report published by Rebuild Consortium	Data analysis reflection on HRH during FHCI: “This was a big [HW] increase on previous years’ trends, even allowing for the fact that some of these new recruits were already working but simply not on payroll”									
Acronyms: FCHI: Free Healthcare Initiative CHC: Community Health Centre CHO[s]: Community Health Officer[s] CHP: Community Health Post				CHW[s]: Community Health Worker[s] GoSL: Government of Sierra Leone HRH: Human Resources for Health HW[s]: Health Worker[s] MCH Aides: Maternal and Child Health Aides			MCHP: Maternal and Child Health Post MoHS: Ministry of Health and Sanitation OOP: Out of Pocket [expenditure] UHW[s]: Unsalaried Health Worker[s]					

### Narrative summary of the findings

#### Charging for care that should be free

The literature contains several openly discussed incidences in which the coping mechanisms of unpaid volunteer health workers are described. Coping mechanisms such as charging for care that should be free, or the selling of medicines that should be free, are most commonly mentioned. Elston et al. (2020) make a direct connection between UHWs and charging for ‘free care’: “Participants also explained that ‘volunteer’ workers would charge for care or services in order to support themselves, and in some cases would continue to do so once salaried in order to ‘make up for’ unpaid years” [61], while Nyhus and Kamara remarked that “Unofficial out-of-pocket payments and a high percentage of the nurses not receiving salary (71%), must also be addressed” [62]. Brooks and Herrick further suggest that at Connaught hospital, Sierra Leone’s largest tertiary referral hospital, fees were levied “at every step of the care pathway” [54]. The authors describe that even items that were donated to improve healthcare provision, such as a blood sugar monitor, can be turned into an income generating opportunity for a staff member, diagnostics can be procured by paying the technician, and for an extra fee, patients can skip the queue [54].

It should be noted that across all of the included literature, a high incidence of charging for care is noted, by salaried and unsalaried staff. Furthermore, it is important to note that not all charging for healthcare is for private gain, as Vernooij et al. [63] are at pains to stress. Their research, also conducted at Connaught hospital, demonstrated that breakdowns in the government’s free healthcare supply chains left lab technicians critically short of reagents and other medical supplies that prevented them from carrying out their duties. Instead of simply not conducting tests, the technicians routinely bought their own supplies and charged patients for tests to recoup their cost.

#### Charging for ‘free’ medicines/record cards

The sale of medicines that should be free to FHCI patients was common even soon after the introduction of free care and free medicines [64, 65]. Disruption to the medical procurement and distribution system during and after the EVD outbreak, and an overall lack of medical supplies since then, seems highlighted in an increasing number of recent studies [62, 66, 67]. The widespread shortage of government-provided medical supplies appears to have created yet another gap in which both opportunists and those who simply want to support patients fill the space. Brooks and Herrick (2019) note that in Connaught hospital “Sierra Leonean nurses went unpaid and sold medicines and supplies ‘from their handbags’” [54], while several articles similarly suggest that health workers purchased medicine and resold them on the wards or to patients at their health centre [62, 63].

#### Mistrust as a result of informal charging

When patients are aware of their entitlement to free care and are consistently denied this, trust in the public health system diminishes. Enria et al’s article on vaccine hesitancy highlights “structural issues that underpin mistrust in the health sector” such as “parents being charged for vaccines and vaccination cards” [68]. Informal charges create uncertainty and unpredictability, making patients unsure if the money they have will be sufficient to cover the cost of the care they need [42, 69, 70]. A lack of trust has previously been related to a reluctance to seek care: It is thought to have undermined the effective control of the West African EVD outbreak, as communities appear to have had little prior trust that health workers would have their best interest at heart [65] and many suspected Ebola to be yet another “political or money making ruse” [71].

Trust is also undermined by a lack of respectful and dignified care, which is often noted as a reason for patients choosing alterative care such as traditional birth attendants or local healers and herbalists [61, 72]. Uncourteous or rude behaviour towards patients can be caused by health workers being stressed, and lacking motivation caused by their unpaid status: Willot et al. (2021) write that health workers’ low morale is exacerbated by perceptions of a lack of recognition of their work by superiors, the management of the hospital or by the MoHS. Health workers in their study commented that they are “just coming to work by the grace of God because there is no salary yet since I started working”, and that low staff morale also affected patients: “…health workers find it difficult to affect their working environment so they take out their frustrations on patients, particularly those who cannot contribute to them getting what they feel that they deserve” [73].

#### Accountability

Absenteeism in the health sector is common in Sierra Leone [40, 41, 54, 65, 70, 74–76]. Holding unsalaried healthcare staff accountable for turning up to work can be challenging, especially when some unpaid staff engage in income generating activities [74] outside of the workplace to make a living. The knock-on effects of the reliance on unaccountable ‘volunteer’ staff are rarely explored. In the context of unmet needs for surgical procedure, Wilson et al. (2022) note the absence of perioperative nurses and scrub nurses, affecting hospital surgical capacity. “Volunteers who are not on the government payroll sometimes join the hospital ranks but present their own set of challenges. Because volunteers have no official compensation structure, they are difficult to hold accountable for the work they perform. Surgical providers find workarounds … One provider, noting that support staff tended to leave by the afternoon, scheduled major surgical cases in the morning” [77].

#### Informal provision of care, private practice and lack of regulation

The literature contains multiple references of informal provision of care, linked, among other things, to the need for UHWs to earn an income. Health workers are documented to offer medical assistance, deliver babies or perform surgeries in patients’ homes, their own homes, makeshift facilities or in health facilities, but ‘off the books’ [74]. A paper by Bakker et al. (2021) addressing the barriers to increasing surgical efficiency, observes that not all surgical procedures are registered, or performed in formal clinics, which makes it hard to assess how many procedures may be taking place. The authors observe that the private sector healthcare providers include “a heterogeneous mix of private and informal facilities and providers” and that ‘informal’ surgical care appears to be linked to “qualified health workers [being] unpaid volunteers for several years before being absorbed on the government payroll, leading to practices to generate informal income” [78]. Sierra Leone’s concentration of highly trained health workers in urban centres, away from the majority rural population, is often linked to the opportunity urban locations provide for private practice [40]. In some hospitals, priority is being given to privately arranged and lucrative care or procedures, according to Nyhus and Kamara, to the detriment of those patients most urgently in need. During training to introduce better triage practices and protocols designed to improve patient outcomes, the authors encountered resistance, as this interfered with opportunities to profit from scheduling the patients according to their ability to pay [62].

### Impact on (and of) national policies and strategies regarding UHWs

#### Overproduction of health workers

The 2017 − 2021 Human Resources for Health Strategy was the first standalone strategy document for Human Resources for Health in Sierra Leone [79]. The strategy, together with the Sierra Leone HRH Country Profile [29], an extensive workforce analysis, and the report on the 2016 HRH summit, attended by Ministry of Health delegates from Liberia, Ethiopia, Zambia, Malawi and Ghana [80], all reference the UHW issue in some detail. The reports highlight the abundance of lower cadre health workers, such as State-Enrolled Community Health Nurses (SECHNs) and Maternal and Child Health Aides (MCHAs), adding that “current production rates far exceed the absorption capacity of MoHS” [29]. After the FHCI introduction, in 2011, it was agreed that a ‘nationally coordinated pre-service training plan’ would be put in place, to govern the training of health workers and to ensure that the ‘production’ of healthcare staff would not outstrip demand [79]. This plan was never implemented. In 2016 there were 25 national health training institutions offering 56 different health programmes, with some training institutions currently operating *without accreditation*” [29] and 11 schools producing roughly 900 total new SECHN graduates per year. In comparison, the single medical officer programme produced approximately 40 new graduates per year [79].

#### Growing number of health facilities, growing demand for healthcare workers

In the context of UHW recruitment, the significant increase in the number of health facilities is noteworthy. Table 5, below, illustrates the number of PHUs that were reported in various MoHS documents between 2012 and 2022 [81–83]. The table shows an increase of 323 PHUs within 10 years, a 31% rise in facility numbers from 2012. This increase in PHUs can only have been possible with a significant number of additional staff, salaried and unsalaried.

**Table 5:** Total number of Peripheral Health Units in Sierra Leone 2012–2022*

	CHCs	CHPs	MCHPs	PHUs total
**2012**	n/a	n/a	n/a	1,040
**2017**	233	329	632	1,184
**2022**	258	433	672	1,363

*Sources: Government of Sierra Leone, Ministry of Health and Sanitation’s National Health Sector Strategic Plan 2010–2015, Joint Programme of Work and Funding; the National Nursing Strategy 2019–2021; and Q2 Health Information Bulletin produced by the MoHS’ Directorate of Policy, Planning and Information

This rapid expansion of PHUs is surprising, in the context of extreme [salaried] health worker shortages and given that the 2017 Service Availability and Readiness Assessment report noted “Eight districts [out of 14] … had a facility density at or above the [WHO] recommended threshold of two facilities per 10 000 population” [84], suggesting that over half of Sierra Leone’s districts had sufficient PHUs. A 2022 qualitative research study on the quality reproductive, maternal, newborn, child, and adolescent healthcare noted that a District Health Management Team (DHMT) member complained of “…the uncoordinated construction of health facilities by politicians ‘to win votes’ also affected the implementation of services in the frontline. This pushed DHMTs to allocate limited resources to the new facilities to satisfy political figures and their followers” [76].

#### UHW issue highlighted and/or included in policy and strategy

The MoHS’ HRH Policy 2017–2021 states that a plan will be developed to absorb unsalaried workers. Actions include the development of a policy to deal with “the remaining unsalaried workers to guide monitoring and regulation of these health workers until all are absorbed, retrained, or removed from facilities” [79]. It contains a detailed plan and costing to recruit UHWs with relevant qualifications to fill HRH vacancies, while others could be offered training to become eligible for recruitment.

Subsequent policy and strategy papers, including the 2021–2025 National Health and Sanitation Policy [47] and the 2021–2025 National Health and Sanitation Strategic Plan [81] both contain references to the unchanged estimate of “approximately 50% healthcare staff who are not on the government payroll” [47]. The strategic plan notes the regulatory challenges the unsalaried workforce brings, as they are “… not subject to the same degree of management and regulation as the formal health workforce” [81]. It further references the need to implement the 2017–2021 HRH Policy [79], however, the objectives under the human resources section appear to focus primarily on renewed fact finding regarding unsalaried workers, stating plans for “comprehensive review of staffing norms, making use as needed of the Workload Indicators of Staffing Needs” and “facility-by-facility health workforce gap analysis” [81].

#### Lack of accurate HRH data undermines MoHS planning

Sierra Leone’s two-tier system with UHWs registered but not on the payroll, poses challenges when the MoHS uses the payroll to determine which quantities of supplies or support should be provided to staff deployed in hospitals and health facilities countrywide. Squire et al. [85, 86] outline how some unsalaried staff were overlooked in Kailahun District during the Ebola epidemic. Their study suggests that when official HRH data is collected, only those on payroll are counted; and add that local health authorities do have the UHW data, though not the data on *unregistered* UHWs [85]. These appear to be staff who attach themselves to health facilities without even local health authority’s knowledge. The authors suggest that “non-registered volunteer staff is of concern” as these completely unregistered individuals had no access to the required biohazard protection measures during the Ebola epidemic: “…such staff… may also be left out when facility requirements for personal protective equipment are being considered. Volunteers will thus be more susceptible to both acquiring and transmitting infectious diseases to co-workers, patients and the community at large” [85].

#### Healthcare financing and UHWs: Performance-Based Finance

Within the literature included in this review, no studies explicitly focused on healthcare financing and UHWs. There were, however, several references to the Performance-Based Financing (PBF) facility, which was implemented from 2011 to 2016. PBF provided health facilities with a certain level of discretionary income, based on each facility’s performance indicators (on ante-natal care, facility deliveries, etc.). PBF provided health facilities with an official income stream, albeit an irregular one, which had been much reduced after the introduction of the free healthcare in 2010. The PBF scheme was launched in 2011, and it “…meant to contribute to the motivation of health workers especially in terms of quality of service and partially compensate for the facilities’ loss of income, while the salary supplementation was seen as compensating the extra workload and to reduce the need to charge fees” [87]. The references to PBF show that the funds provided payment for the growing number of UHWs. There was even a “widespread perception of the PBF payment as a sort of salary [rather than an incentive] for staff, in particular for the unsalaried volunteers” [87]. Several papers stress that the funding was often a lifeline for unsalaried workers: “sharing [PBF funding] practices, highlighting the existence of team spirit within facilities, were found, in particular in health centres with fewer staff.” [75]. An improved PBF scheme was meant to have been launched after the first iteration stalled during the Ebola outbreak. While the 2021–2025 Health Strategic Plan [81] mentions the introduction of a new PBF scheme, so far, no scheme has been launched.

## Discussion

This scoping review of evidence about the impact of UHWs demonstrates that the reliance, to such a large extent, on an unsalaried work force undermines the equitable delivery of healthcare. The recruitment of health workers on an unsalaried basis also violates their right to a wage [88]. Even though no studies exist that have been focused exclusively on Sierra Leone’s UHWs, the review provides evidence that highlights a range of unexpected impacts that UHW may have on the health system’s ability to provide equitable care.

The findings presented in this paper demonstrate that a parallel system of employment for qualified but unsalaried healthcare providers, has existed for decades within formal state-run health facilities. This appears to be an ignored or acceptable ‘make do’ solution within a health system characterised by underinvestment in primary healthcare and an oversupply of low-cadre health workers. It is important to discuss the impact that each of the identified themes presents.

### Charging for care and medicine that should be free

The literature suggests that in Sierra Leone, charging the ‘free healthcare target populations’ for ‘free care’ never completely ceased, even in the weeks and months after the FCHI introduction [53, 69]. One study in 2012 by Stevenson et al. [64] estimated that approximately 20% of FHCI-eligible patients were charged, while Jofre-Bonnet et al. 2016 study shows that “96% of households responded they paid for FHC services even though they were meant to be free” [50]. The 2021–2025 Health Policy and National Health Strategic Plan highlight that Sierra Leone’s households out of pocket (OOP) expenditures are among the highest in sub-Saharan Africa, at 61% [47, 81]. Charging for care that should be free, or overcharging for care and medicines is not exclusively linked to UHW; salaried workers may engage in similar informal practices to boast their salaries [89]. The study by Oyerinde et al., 2011 suggests a correlation between out-of-pocket expenditure to the low utilization of services in Sierra Leone [69]. Informal increases in the cost of care and medication are relatively common in Sub-Saharan Africa (SSA), where the ‘supply-side factors’ of informal charges, identified by Kabia et al. (2021) as “associated with inadequate funding of the health sector, limited transparency and accountability and low/irregular remuneration of staff” are widespread [90]. Studies focused on corruption in the health sector in Tanzania, Cameroon and Nigeria, all identify informal charges for care and medication as one of the most common forms of corruption [91–93]. In low-income settings, informal charges can limit access to healthcare, and where high levels of mortality and morbidity have treatable and avoidable causes, it can be assumed that financial barriers increase maternal and child deaths [15]. Thaddeus and Maine’s ‘three delays model’ (1984) that explains the key factors that contribute to maternal mortality, highlight the role of cost as a significant barrier to the first delay; the decision to seek care [94]. Levesque et al’s (2013) framework on access to healthcare includes ‘affordability’ as one of the five dimensions of ‘accessibility’, which they describe as the “opportunity to identify healthcare needs, to seek healthcare services, to reach, to obtain or use health care services, and to actually have a need for services fulfilled” [95].

### Mistrust

In Sierra Leone, trust in healthcare providers has been undermined by decades of informal charges for care and services that should have been free to certain sections of the population. A lack of trust in public healthcare providers reduces healthcare uptake [65], decreases immunisation coverage [68] and is thought to have contributed to the rapid spread of the Ebola virus during the 2014–2016 EVD epidemic in West Africa [96–98]. While trust can be (re)built in a LMIC healthcare setting [4, 99, 100], this can only happen when the underlying causes of why trust was undermined have been resolved.

### Accountability

The concept of accountability, in a health sector context, presupposes that an employee can be held to account for behaving in a way that conforms with their contractual obligations, which should include both a job description and salary entitlements [101]. However, in the case of UHWs, the grounds on which such employees can be held accountable to work are much less clear. In both Sierra Leone and Liberia, medical and nursing school graduates tend to be offered a ‘pre-employment’ deal whereby health workers enter a workplace with the assumption that they will eventually be entered onto the payroll system [24, 26]. In Liberia, such ‘contract’ employees receive some payment, even though it can be irregular or insecure [26]. Thus, while it can be argued that some form of contract exists between the unsalaried or contract employee and the health service, it can be contended that it is morally wrong to enforce it. The use of internships, apprenticeships, and other work experience schemes are relatively common in the Global North, where such schemes have increasingly become a prerequisite for young professionals to obtaining decent work [102, 103]. Increasingly such mechanisms have been criticised for “being used as a way of obtaining cheap labour or replacing existing workers” [103].

### Informal provision of care, private practice, and lack of regulation

The literature on Sierra Leone’s UHWs makes references to health workers, especially those in higher cadres, engaging in private practice to supplement their income [74]. Some ‘private’ surgical care appears to be provided within public hospitals [73], where “financial incentives exist not to record surgical activities in hospital logbooks” [78]. While there are some established private-for-profit and private-not-for-profit healthcare providers in Sierra Leone, other private providers operate outside of such establishments. The literature on regulating private healthcare practice in LMIC settings is limited [104–106]. Health workers engaging in *informal private practice* is a critical issue, not only in the context of ‘dual practice’ – leading to absenteeism of health workers who devote time to see private patients [107], but also with regards to protecting the public from unregulated medical practice. The descriptions of informal private practice resulting in “moribund patients” arriving in public health facilities, demonstrate the health risks these alternative income streams pose to the public [78]. If Sierra Leone’s recruitment of UHWs leads, as some evidence suggests, to an increase in dangerous private provision of healthcare, it would be yet another reason why this practice should be curtailed.

### Policies and strategies in relation to UHWs, including staff production

Tackling the UHW issue in Sierra Leone should start with implementing the existing policy commitments; addressing the health workforce planning and the regulation of training institutions to close the gap between the number of graduates from medical and nursing schools and the demand, based on available paid posts, with a greater focus on producing higher-cadre graduates. Sierra Leone’s 2021–2025 National Nursing and Midwifery Strategic Plan notes that “there have been discussions and plans to abolish the SECHN in the near future” [82], which would be an important first step. Sierra Leone *health worker density* is currently 6.4 per 10,000 population [81]. This international measurement takes into account only higher cadre health workers such as doctors, and midwives and nurses who have completed their professional training (i.e. not the auxiliary cadres such as SECHNs and MCH Aides). Sierra Leone needs significantly more high cadre health workers to meet the average health worker density for the WHO Africa region, which is 15.5 per 10,000 population, well below the WHO recommended 22.6 per 10,000 population minimum [108]. The 2021–2025 National Health Strategic Plan aims to bring the country’s health worker density to 45 per 10,000 population by 2025 [81].

To achieve its 2021–2025 HRH policy pledges, Sierra Leone will need significant political will to commit the required budget, as well as donor support. This would not only mitigate the significant negative impact that the reliance on an unsalaried health workforce has on healthcare delivery, it would also contribute to Sierra Leone’s policy commitment to achieve Universal Health Coverage by 2030 [3, 47, 109].

### Health financing

The literature included in this scoping review contains few references to one of the key underlying factors of the UHW issue: healthcare financing. Understanding some of the key challenges regarding Sierra Leone’s health financing allows for a greater insight into why the country continues to rely heavily on UHWs. A piece in the Lancet in the early stages of the EVD epidemic highlighted the International Monetary Fund’s *then* 19-year-long support for Sierra Leone, which comes with stringent micro-economic conditions, including keeping the country’s wage bill in check [110, 111]. The IMF was criticised for requiring caps on the public-sector wage bill, limiting funds that are available to hire or adequately remunerate health-care professionals; such limits were thought to be set without consideration of the impact on priority areas such as health.

While aid conditionality is one issue that undermines the availability of sufficient funding for a payroll expansion; unproductive and irregular healthcare spending seem to further compound these challenges. A 2021 World Bank Public Expenditure Review of Sierra Leone’s health sector reveals that public health spending in Sierra Leone is higher than in its West African sub-regional neighbours, but health outcomes are lower. Government expenditure on health was 1.56% of GDP, and in 2019, the health sector received the second highest allocation of funding, at 6.49% of the overall budget, second only to education [112]. The same report revealed that 73% of all health funding was spent on administrative services. Secondary and tertiary care services received 12% of the funds, leaving just 3% for primary health care. The report summarises key issues the authors encountered during their review, suggesting Sierra Leone’s health sector suffers from:

unpredictable levels of health expenditure; low capital expenditure, resulting in inadequate availability of health infrastructure; high expenditure on personnel emoluments, crowding out spending on goods and services such as essential drugs and medical supplies; most capital expenditure going toward transfers to other agencies of general government for purposes which are unclear; … budgetary allocations to Local Councils for primary health care delivery not tied to performance targets; little or no spending dedicated to infectious diseases due to unsustainable reliance on donor support; high budget execution rate not commensurate with performance in terms of health outcomes; weak district-level public financial management capabilities; and uneven distribution of healthcare resources across the country.

### Limitations of this review

The authors of this scoping review have taken the unorthodox approach of identifying, mapping, reporting and discussing [56] the characteristics and impacts of our emerging concept, UHWs in Sierra Leone, that could be found *within* the existing literature, grey literature and policy documentation related to healthcare in that country. While rigorous searches were conducted to capture all evidence that could be found about the topic, this review is limited by the fact that no studies have so far been focused solely on UHWs in Sierra Leone. As a result, this study presents a relatively narrow summing up of observations regarding UHWs, which were made by authors whose emphasis was on a related health topic. Despite this limitation, there was sufficient evidence to allow this scoping review to be compiled, which was demonstrated by the fact that several themes could clearly be discerned from the available material. This scoping review suggests that more research is needed to improve our understanding of UHWs’ coping mechanisms and of their impact on access to healthcare for all Sierra Leoneans.

## Conclusion

This scoping review has compiled all available evidence to demonstrate that the significant and continued presence of UHWs within Sierra Leone’s health system undermines the delivery of quality and equitable healthcare. The key themes that emerged point to a series of unintended consequences that can be associated with the heavy reliance on UHWs; UHW appear to be linked to informal charging for care and medication (including charging pregnant women and children under five entitled to free care), leading to some mistrust of health workers. UHWs were shown to lack accountability and sometimes engage in unregulated private practice that can lead to unfavourable health outcomes. Whilst the MoHS has acknowledged the health sector’s reliance on unsalaried health workforce and has previously published policy commitments to address the issue, little has changed since the publication in 2016 that demonstrated that almost half of all of Sierra Leone’s health workers were unsalaried.

### Electronic supplementary material

Below is the link to the electronic supplementary material.

Supplementary Material 1

Supplementary Material 2

## Data Availability

The additional materials supporting the conclusions of this article are available in the Open Science Framework repository, in 10.17605/OSF.IO/X2G6P.

## List of Abbreviations

CHC: Community Health Centre
CHO: Community Health Officer
CHP: Community Health Post
CHW: Community Health Worker
DHMT: District Health Management Teams
EVD: Ebola Virus Disease
FHCI: Free Healthcare Initiative
GoSL: Government of Sierra Leone
HRH: Human Resources for Health
HW: Health Worker
MCHA: Maternal and Child Health Aide
MCHP: Maternal and Child Health Post
MoHS: Ministry of Health and Sanitation
OOP: Out of pocket [expenditures]
PBF: Performance-Based Finance
PHU: Peripheral Health Unit
PPE: Personal Protection Equipment
SARA: Service Accessibility and Readiness Assessment
SBA: Skilled Birth Attendant
SECHN: State-Enrolled Community Health Nurse
UHW: Unsalaried Health Worker

## References

[1] Boniol M, Kunjumen T, Nair TS, Siyam A, Campbell J, Diallo K (2022). The global health workforce stock and distribution in 2020 and 2030: a threat to equity and ‘universal’ health coverage?. BMJ Glob Health.

[2] Yeates N, Pillinger J (2019). International health worker migration and recruitment: global governance, politics and policy.

[3] Reid M, Gupta R, Roberts G, Goosby E, Wesson P. Achieving Universal Health Coverage (UHC): Dominance analysis across 183 countries highlights importance of strengthening health workforce. Mathur MR, editor. PLOS ONE. 2020;15(3):e0229666. 10.1371/journal.pone.0229666 PMC7055867 32130241

[4] Schwarz D, Hirschhorn LR, Kim JH, Ratcliffe HL, Bitton A (2019). Continuity in primary care: a critical but neglected component for achieving high-quality universal health coverage. BMJ Glob Health.

[5] Darzi A, Evans T (2016). The global shortage of health workers provides an opportunity to transform care. The Lancet.

[6] Liu JX, Goryakin Y, Maeda A, Bruckner T, Scheffler R. Global Health Workforce Labor Market Projections for 2030. 10.1186/s12960-017-0187-2 PMC5291995 28159017

[7] Asamani JA, Akogun OB, Nyoni J, Ahmat A, Nabyonga-Orem J, Tumusiime P (2019). Towards a regional strategy for resolving the human resources for health challenges in Africa. BMJ Glob Health.

[8] World Health Organisation (2022). WORKING FOR HEALTH 2022–2030 action plan.

[9] Ahmed S, Chase LE, Wagnild J, Akhter N, Sturridge S, Clarke A (2022). Community health workers and health equity in low- and middle-income countries: systematic review and recommendations for policy and practice. Int J Equity Health.

[10] Asamani JA, Kigozi J, Sikapande B, Christmals CD, Okoroafor SC, Ismaila H (2022). Investing in the health workforce: fiscal space analysis of 20 countries in East and Southern Africa, 2021–2026. BMJ Glob Health.

[11] .: ; 2023. WHO Regional Office for Africa. Analysis of the nature and contribution of innovative health financing mechanisms in the WHO African Region, Brazzaville. Congo: WHO Regional Office for Africa; 2023.

[12] Piatti-Funfkirchen M, Barroy H, Pivodic F, Margini F. Budget Execution in Health: Concepts, Trends and Policy Issues [Internet]. World Bank; 2021 [cited 2023 Oct 11]. Available from: http://elibrary.worldbank.org/doi/book/10.1596/36583.

[13] Musiega A, Tsofa B, Nyawira L, Njuguna RG, Munywoki J, Hanson K (2023). Examining the influence of budget Execution processes on the efficiency of county health systems in Kenya. Health Policy Plan.

[14] Hussmann K. Addressing corruption in the health sector.

[15] Vian T (2020). Anti-corruption, transparency and accountability in health: concepts, frameworks, and approaches. Glob Health Action.

[16] World Health Organisation (2020). Findings from a rapid review of literature on ghost workers in the health sector: towards improving detection and prevention.

[17] Manyerere DJ, Mpambije CJ. Rethinking the terrain of ghost workers in Sub-saharan Africa: a manifestation of the Governance Status and Poverty Dilemma. 2022;20(1).

[18] World Health Organization. Global strategy on human resources for health: workforce 2030 [Internet]. Geneva: World Health Organization. ; 2016 [cited 2023 Sep 15]. 64 p. Available from: https://apps.who.int/iris/handle/10665/250368.

[19] Adovor E, Czaika M, Docquier F, Moullan Y (2021). Medical brain drain: how many, where and why?. J Health Econ.

[20] Muula AS (2023). The paradox of Malawi’s health workforce shortage: pragmatic and unpopular decisions are needed. Malawi Med J.

[21] Hutchinson E, Hansen KS, Sanyu J, Amonya LP, Mundua S, Balabanova D (2023). Is it possible for drug shops to abide by the formal rules? The structural determinants of community medicine sales in Uganda. BMJ Glob Health.

[22] Hutchinson E, Mundua S, Ochero L, Mbonye A, Clarke SE (2022). Life in the buffer zone: social relations and surplus health workers in Uganda’s medicines retail sector. Soc Sci Med.

[23] Jansen C, Codjia L, Dieleman M, Lamine Y, Cometto G. Realizing universal health coverage for maternal health services in the Republic of Guinea: the use of workforce projections to design health labor market interventions. Risk Manag Healthc Policy. 2014;219. 10.2147/RMHP.S46418 PMC4243577 25429245

[24] Narayan V, John-Stewart G, Gage G, O’Malley G. If I had known, I would have applied: poor communication, job dissatisfaction, and attrition of rural health workers in Sierra Leone. Hum Resour Health. 2018;16(1). 10.1186/s12960-018-0311-y PMC6154815 30249253

[25] Govindaraj R, Herbst CH, Ajumobi O, Rockmore C, Driss M, El Idrissi ZE et al. POST-EBOLA HEALTH SYSTEMS From Response to Resilience in Guinea, Liberia, and Sierra Leone.

[26] FHI360. Understanding Liberian Healthcare Worker interactions with Payment systems, Mobile Phones, and Financial Behavior: an Ethnographic Study. USAID; 2016.

[27] Kolie D, Van De Pas R, Delamou A, Dioubaté N, Beavogui FT, Bouedouno P (2021). Retention of healthcare workers 1 year after recruitment and deployment in rural settings: an experience Post-ebola in five health districts in Guinea. Hum Resour Health.

[28] van de Pas R, Kolie D, Delamou A, Van Damme W (2019). Health workforce development and retention in Guinea: a policy analysis Post-ebola. Hum Resour Health.

[29] Government of Sierra Leone. Ministry of Health and Sanitation. Human Resources for Health, Sierra Leone Country Profile; 2016.

[30] World Bank. World Bank data. 2022. Total population, Sierra Loene. Available from: https://data.worldbank.org/indicator/SP.POP.TOTL?locations=SL.

[31] Barr A, Garrett L, Marten R, Kadandale S (2019). Health sector fragmentation: three examples from Sierra Leone 16 studies in Human Society 1605 policy and administration. Glob Health.

[32] UN Inter-agency Group for Child Mortality Estimation. CHILD MORTALITY AND STILLBIRTH ESTIMATES [Internet]. 2023. Available from: https://childmortality.org/data.

[33] Carshon-Marsh R, Aimone A, Ansumana R, Swaray IB, Assalif A, Musa A (2022). Child, maternal, and adult mortality in Sierra Leone: nationally representative mortality survey 2018–20. Lancet Glob Health.

[34] Midtgaard Eriksen C, Lauridsen Kujabi M, Sulaiman Kanu A, Gulis G (2021). Health Perceptions in Relation to Child Health and Mortality in a rural context, Sierra Leone: a mixed method study. Int J Environ Res Public Health.

[35] Statistics Sierra Leone Stats SL and ICF (2020). Sierra Leone Demographic and Health Survey 2019 [FR365].

[36] Integrated African Health Observatory, WHO. Maternal mortality: The urgency of a systemic and multisectoral approach in mitigating maternal deaths in Africa [Internet]. 2023. Available from: https://files.aho.afro.who.int/afahobckpcontainer/production/files/iAHO_Maternal_Mortality_Regional_Factsheet.pdf.

[37] Elston JWT, Danis K, Gray N, West K, Lokuge K, Black B et al. Maternal health after Ebola: unmet needs and barriers to healthcare in rural Sierra Leone. Health Policy Plan. 2019;czz102. 10.1093/heapol/czz102 31697378

[38] Raven J, Wurie H, Witter S. Health workers’ experiences of coping with the Ebola epidemic in Sierra Leone’s health system: a qualitative study. BMC Health Serv Res. 2018;18(1). 10.1186/s12913-018-3072-3 PMC5887191 29622025

[39] Jamison DT, Breman JG, Measham AR. Disease control priorities in developing countries. Second Edition. New York: Oxford University Press and the World Bank; 2006. 21250309

[40] Bertone MP, Witter S. The development of HRH policy in Sierra Leone, 2002–2012 – report on key informant interviews. 2013;(December):2002–12.

[41] Witter S, Brikci N, Harris T, Williams R, Keen S, Mujica A (2018). The free healthcare initiative in Sierra Leone: evaluating a health system reform, 2010-2015. Int J Health Plann Manage.

[42] Amnesty International (2009). Out of Reach: the cost of maternal health in Sierra Leone.

[43] Government of Sierra Leone. Sierra Leone Government directs that all health facilities should provide free health care for Ebola survivors [Press Release] [Internet]. COCORIOKO. 2016 [cited 2023 May 25]. Available from: https://cocorioko.net/sierra-leone-government-directs-that-all-health-facilities-should-provide-free-health-care-for-ebola-survivors/.

[44] Denney L, Mallet R (2014). Mapping Sierra Leone ’ s plural health system and how people navigate it. Secure Livelihoods Res Consort.

[45] Liquat S, Ferry J (2011). Free health care six months on: what does it mean for child health in Northern Bombali?.

[46] Edoka I, Ensor T, McPake B, Amara R, Tseng FM, Edem-Hotah J (2015). Free health care for under-fives, expectant and recent mothers? Evaluating the impact of Sierra Leone’s free health care initiative. Health Econ Rev.

[47] Government of Sierra Leone., Ministry of Health and Sanitation. NATIONAL HEALTH AND SANITATION POLICY 2021. 2021.

[48] Srivastava V, Larizza M. Decentralization in Postconflict Sierra Leone: The Genie Is Out of the Bottle. In: Yes Africa can: Success stories from a dynamic continent. 2011. p. 141–54.

[49] World Bank Poverty Reduction and Economic Management Africa Region. Decentralization, Accountability and Local Services In Sierra Leone: Situation Analysis, Key Challenges and Opportunities For Reform. 2014.

[50] Jofre-Bonet M, Kamara J, Mesnard A. Corruption and Health Insurance for the Informal Sector in Sierra Leone. 2021.

[51] Walsh S, Johnson O. Getting to zero: a doctor and a diplomat on the Ebola frontline. Bloomsbury Publishing; 2018.

[52] Bertone MP, Witter S (2015). An exploration of the political economy dynamics shaping health worker incentives in three districts in Sierra Leone. Soc Sci Med.

[53] Amnesty International (2011). At a crossroads. Sierra Leone’s Free Health Care Policy.

[54] Brooks A, Herrick C (2019). Bringing relational comparison into development studies: global health volunteers’ experiences of Sierra Leone. Prog Dev Stud.

[55] Peters MDJ, Godfrey CM, Khalil H, McInerney P, Parker D, Soares CB (2015). Guidance for conducting systematic scoping reviews. Int J Evid Based Healthc.

[56] Arksey H, O’Malley L (2005). Scoping studies: towards a methodological framework. Int J Soc Res Methodol.

[57] Levac D, Colquhoun H, O’Brien KK (2010). Scoping studies: advancing the methodology. Implement Sci.

[58] Peters MDJ, Marnie C, Tricco AC, Pollock D, Munn Z, Alexander L (2020). Updated methodological guidance for the conduct of scoping reviews. JBI Evid Synth.

[59] Peters MDJ, Marnie C, Colquhoun H, Garritty CM, Hempel S, Horsley T (2021). Scoping reviews: reinforcing and advancing the methodology and application. Syst Rev.

[60] Tricco AC, Erin L, Wasifa Z (2018). PRISMA Extension for scoping reviews (PRISMA-ScR): Checklist and explanations. Ann Intern Med.

[61] Elston JWT, Danis K, Gray N, West K, Lokuge K, Black B (2020). Maternal health after Ebola: unmet needs and barriers to healthcare in rural Sierra Leone. Health Policy Plan.

[62] Nyhus HB, Kamara MM (2017). Quality improvement in emergency service delivery: Assessment of knowledge and skills amongst emergency nurses at Connaught Hospital, Sierra Leone. Afr J Emerg Med Rev Afr Med Urgence.

[63] Vernooij E, Koker F, Street A (2022). Responsibility, repair and care in Sierra Leone’s health system. Soc Sci Med.

[64] Stevenson D, Kinyeki C, Wheeler M. Evaluation of DFID Support to Healthcare Workers Salaries in Sierra Leone [Internet]. Available from: www.hlsp.org.

[65] Pieterse P, Lodge T (2015). When free healthcare is not free. Corruption and mistrust in Sierra Leone’s primary healthcare system immediately prior to the Ebola outbreak. Int Health.

[66] Frankfurter R (2019). Conjuring Biosecurity in the Post-ebola Kissi Triangle: the Magic of Paperwork in a Frontier Clinic. Med Anthropol Q.

[67] Miller NP, Milsom P, Johnson G, Bedford J, Kapeu AS, Diallo AO et al. Community health workers during the Ebola outbreak in Guinea, Liberia, and Sierra Leone. J Glob Health. 2018;8(2). 10.7189/jogh-08-020601 PMC6030670 30023054

[68] Enria L, Bangura JS, Kanu HM, Kalokoh JA, Timbo AD, Kamara M et al. Bringing the social into vaccination research: community-led ethnography and trust-building in immunization programs in Sierra Leone. PLoS ONE. 2021;16(10 October). 10.1371/journal.pone.0258252 PMC8535180 34679104

[69] Oyerinde K, Harding Y, Amara P, Kanu R, Shoo R, Daoh K (2011). The status of maternal and newborn care services in Sierra Leone 8 years after ceasefire. Int J Gynecol Obstet.

[70] Witter S, Mujica AM, Jones A, Leigh B. The Sierra Leone Free Health Care Initiative (FHCI): process and effectiveness review Prevention of maternal mortality inSierra Leone View project FEMHealth View project. 2016;(April). Available from: https://www.researchgate.net/publication/303234918.

[71] Richards P, Mokuwa E, Welmers P, Maat H, Beisel U. Trust, and distrust, of Ebola Treatment Centers: a case-study from Sierra Leone. PLoS ONE. 2019;14(12). 10.1371/journal.pone.0224511 PMC6886773 31790420

[72] Dorwie FM, Pacquiao DF (2014). Practices of Traditional Birth attendants in Sierra Leone and perceptions by mothers and Health professionals Familiar with their care. J Transcult Nurs.

[73] Willott C, Boyd N, Wurie H, Smalle I, Kamara TB, Davies JI (2021). Staff recognition and its importance for surgical service delivery: a qualitative study in Freetown, Sierra Leone. Health Policy Plan.

[74] Bertone MP, Lagarde M (2016). Sources, determinants and utilization of health workers’ revenues: evidence from Sierra Leone. Health Policy Plan.

[75] Bertone MP. Performance-Based Financing in the context of the ‘complex remuneration’ of Health Workers. 10.1186/s12913-016-1546-8 PMC4952280 27435164

[76] Tengbeh AF, Bertone MP, Witter S, Enria L. Implementation of the Reproductive, maternal, newborn, child, & Adolescent Health Policy in Sierra Leone. Rebuild for Resilience; 2022.

[77] Wilson D (2015). Inside an Ebola Treatment Unit: a nurse’s report. AJN Am J Nurs.

[78] Bakker J, Van Duinen AJ, Nolet WWE, Mboma P, Sam T, Van Den Broek A et al. Barriers to increase surgical productivity in Sierra Leone: a qualitative study. BMJ Open. 2021;11(12). 10.1136/bmjopen-2021-056784 PMC8693091 34933865

[79] Government of Sierra Leone., Ministry of Health and Sanitation. Human Resources for Health Strategy 2017–2021. 2017.

[80] Government of Sierra Leone (2016). Ministry of Health and Sanitation. HUMAN RESOURCES FOR HEALTH SUMMIT, 2–3 JUNE 2016.

[81] Government of Sierra Leone., Ministry of Health and Sanitation. NATIONAL HEALTH SECTOR STRATEGIC PLAN 2021–2025. 2021.

[82] Government of Sierra Leone., Ministry of Health and Sanitation. NATIONAL NURSING AND MIDWIFERY STRATEGIC PLAN 2 0 1 9 – 2 0 2 3. 2019.

[83] Directorate of Policy, Planning and Information (DPPI)., Ministry of Health and Sanitation (MOHS). Better Information, Better Planning, Better Health. Health Information Bulletin, Q2 2022. 2022.

[84] Government of Sierra Leone., Ministry of Health and Sanitation. The Sierra Leone Service Availability and Readiness Assessment (SARA) 2017. 2017.

[85] Squire JS, Hann K, Denisiuk O, Kamara M, Tamang D, Zachariah R (2017). The Ebola outbreak and staffing in public health facilities in rural Sierra Leone: who is left to do the job?. Public Health Action.

[86] Squire JS, Hann K, Denisiuk O, Zachariah R. Staffing in public health facilities after the ebola outbreak in rural Sierra Leone: How much has changed? F1000Research. 2020;8. 10.12688/f1000research.18566.1 PMC7047906 32148756

[87] Bertone MP, Wurie H, Samai M, Witter S (2018). The bumpy trajectory of performance-based financing for healthcare in Sierra Leone: Agency, structure and frames shaping the policy process. Glob Health.

[88] International Labor Organisation. Social, Protection S, Development, Right to Development. Statement by, Director-General ILO. Gilbert F. Houngbo, at the Human Rights 75 - September Thematic Spotlight Discussion co organized by the ILO and the UN Human Rights Office. [Internet]. 2023. Available from: https://www.ilo.org/global/about-the-ilo/how-the-ilo-works/ilo-director-general/statements-and-speeches/WCMS_895511/lang--en/index.htm.

[89] Onwujekwe O, Agwu P, Orjiakor C, McKee M, Hutchinson E, Mbachu C (2019). Corruption in Anglophone West Africa health systems: a systematic review of its different variants and the factors that sustain them. Health Policy Plan.

[90] Kabia E, Goodman C, Balabanova D, Muraya K, Molyneux S, Barasa E (2021). The hidden financial burden of healthcare: a systematic literature review of informal payments in Sub-saharan Africa. Wellcome Open Res.

[91] Binyaruka P, Balabanova D, McKee M, Hutchinson E, Andreoni A, Ramesh M (2021). Supply-side factors influencing informal payment for healthcare services in Tanzania. Health Policy Plan.

[92] Kankeu HT, Boyer S, Fodjo Toukam R, Abu-Zaineh M (2016). How do supply-side factors influence informal payments for healthcare? The case of HIV patients in Cameroon. Int J Health Plann Manage.

[93] Onwujekwe O, Orjiakor CT, Hutchinson E, McKee M, Agwu P, Mbachu C et al. Where do we start? Building Consensus on drivers of Health Sector Corruption in Nigeria and ways to address it. Int J Health Policy Manag. 2019;1. 10.15171/ijhpm.2019.128 PMC7444438 32613800

[94] Thaddeus S, Maine D (1994). Too far to walk: maternal mortality in context. Soc Sci Med.

[95] Levesque JF, Harris MF, Russell G (2013). Patient-centred access to health care: conceptualising access at the interface of health systems and populations. Int J Equity Health.

[96] Blair RA, Morse BS, Tsai LL (2017). Public health and public trust: Survey evidence from the Ebola Virus Disease epidemic in Liberia. Soc Sci Med.

[97] Nuriddin A, Jalloh MF, Meyer E, Bunnell R, Bio FA, Jalloh MB (2018). Trust, fear, stigma and disruptions: community perceptions and experiences during periods of low but ongoing transmission of Ebola virus Disease in Sierra Leone, 2015. BMJ Glob Health.

[98] Piot P (2014). Ebola’s perfect Storm. Science.

[99] Ezumah N, Manzano A, Ezenwaka U, Obi U, Ensor T, Etiaba E (2022). Role of trust in sustaining provision and uptake of maternal and child healthcare: evidence from a national programme in Nigeria. Soc Sci Med.

[100] Nganga SW, Otieno NA, Adero M, Ouma D, Chaves SS, Verani JR (2019). Patient and provider perspectives on how trust influences maternal vaccine acceptance among pregnant women in Kenya. BMC Health Serv Res.

[101] Brinkerhoff DW (2004). Accountability and health systems: toward conceptual clarity and policy relevance. Health Policy Plan.

[102] McDonald P, Grant-Smith D, Wilkinson A, Barry M (2020). Unpaid work experience and interships: a growing and contested feature of of the future of work. The future of work and employment.

[103] Stewart A, Owens R, O’Higgins N, Hewitt A, editors. Internships, Employability and the Search for Decent Work Experience [Internet]. Edward Elgar Publishing; 2021 [cited 2023 Sep 22]. Available from: https://www.elgaronline.com/view/edcoll/9781800885035/9781800885035.xml.

[104] Hipgrave DB, Hort K (2014). Dual practice by doctors working in South and East Asia: a review of its origins, scope and impact, and the options for regulation. Health Policy Plan.

[105] Sheikh K, Saligram P, Prasad LE. Mapping the regulatory architecture for health care delivery in mixed health systems in low- and middle-income countries.

[106] Zainuddin NA, Noh KM, Abdullah Z. Challenges in regulating private primary Health Care in Malaysia: perceptions from Key informants. Nur Azmiah Zainuddin1, Kamaliah Mohd Noh2, Zalilah Abdullah1, Nur Hidayati Abdul Halim1, Rima Marhayu Abdul Rashid3, Ainul Nadziha Mohd. Hanafiah, nor Idawaty Ibrahim4, Safiee Ismail4. Asian J Med Health Sci. 2022;5(1).

[107] Kiwanuka SN, Rutebemberwa E, Nalwadda C, Okui O, Ssengooba F, Kinengyere AA et al. Interventions to manage dual practice among health workers. Cochrane Effective Practice and Organisation of Care Group, editor. Cochrane Database Syst Rev [Internet]. 2011 Jul 6 [cited 2023 Sep 22]; 10.1002/14651858.CD008405.pub2. 10.1002/14651858.CD008405.pub2 PMC6791302 21735429

[108] Ahmat A, Okoroafor SC, Kazanga I, Asamani JA, Millogo JJS, Illou MMA (2022). The health workforce status in the WHO African Region: findings of a cross-sectional study. BMJ Glob Health.

[109] Noya F, Carr S, Freeman K, Thompson S, Clifford R, Playford D. Strategies to facilitate Improved Recruitment, Development, and Retention of the Rural and Remote Medical Workforce: a scoping review. Int J Health Policy Manag. 2021;1. 10.34172/ijhpm.2020.27 PMC7947704 32610716

[110] Kentikelenis A, King L, McKee M, Stuckler D (2015). The International Monetary Fund and the Ebola outbreak. Lancet Glob Health.

[111] Stubbs T, Kentikelenis A, Stuckler D, McKee M, King L (2017). The impact of IMF conditionality on government health expenditure: a cross-national analysis of 16 west African nations. Soc Sci Med.

[112] World Bank. SIERRA LEONE, PUBLIC EXPENDITURE REVIEW. 2021: Improving Quality of Public Expenditure in Health. 2021.

