# Supplementary material for: Unsalaried health workers in Sierra Leone: a scoping review of the literature to establish their impact on healthcare delivery
Source: Int J Equity Health. 2023 Dec 9;22:255. doi: 10.1186/s12939-023-02066-3 (PMC10709924; doi:10.1186/s12939-023-02066-3)
Supplement: Supplementary file 2 — Supplementary Material 2 [file 12939_2023_2066_MOESM2_ESM.pdf]

1. Amnesty International. Out of Reach: The Cost of Maternal Health in Sierra Leone. London: Amnesty International; 2009.

2. Bakker J, van Duinen AJ, Nolet WWE, Mboma P, Sam T, van den Broek A, et al. Barriers to increase surgical productivity in Sierra Leone: a qualitative study. BMJ Open. 2021 Dec;11(12):e056784.

3. Bertone MP, Lagarde M. Sources, determinants and utilization of health workers' revenues: evidence from Sierra Leone. Health Policy Plan. 2016 Oct;31(8):1010–9.

4. Bertone MP, Lagarde M, Witter S. Performance-based financing in the context of the complex remuneration of health workers: Findings from a mixed-method study in rural Sierra Leone. BMC Health Services Research. 2016;16(1):1–10.

5. Bertone MP, Witter S. The development of HRH policy in Sierra Leone, 2002-2012 – report on key informant interviews. 2013;(December):2002–12.

6. Bertone MP, Witter S. An exploration of the political economy dynamics shaping health worker incentives in three districts in Sierra Leone. Social Science & Medicine. 2015 Sep;141:56–63.

7. Brooks A, Herrick C. Bringing relational comparison into development studies: Global health volunteers' experiences of Sierra Leone. Progress in Development Studies. 2019 Apr;19(2):97–111.

8. Dorwie FM, Pacquiao DF. Practices of Traditional Birth Attendants in Sierra Leone and Perceptions by Mothers and Health Professionals Familiar With Their Care. Journal of Transcultural Nursing. 2014;25(1):33–41.

9. Elston JWT, Danis K, Gray N, West K, Lokuge K, Black B, et al. Maternal health after Ebola: unmet needs and barriers to healthcare in rural Sierra Leone. Health Policy and Planning. 2019 Nov 7;czz102.

10. Enria L, Bangura JS, Kanu HM, Kalokoh JA, Timbo AD, Kamara M, et al. Bringing the social into vaccination research: Community-led ethnography and trust-building in immunization programs in Sierra Leone. Newman PA, editor. PLoS ONE. 2021 Oct 22;16(10):e0258252.

11. Frankfurter R. Conjuring Biosecurity in the Post-Ebola Kissi Triangle: The Magic of Paperwork in a Frontier Clinic. Medical Anthropology Quarterly. 2019;33(4):517–38.

12. Government of Sierra Leone, Ministry of Health and Sanitation. HUMAN RESOURCES FOR HEALTH SUMMIT, 2-3 JUNE 2016, FREETOWN, SIERRA LEONE. 2016.

13. Government of Sierra Leone, Ministry of Health and Sanitation. Human Resources for Health, Sierra Leone Country Profile. 2016.

14. Government of Sierra Leone, Ministry of Health and Sanitation. Human Resources for Health Strategy 2017-2021. 2017.

15. Government of Sierra Leone, Ministry of Health and Sanitation. The Sierra Leone Service Availability and Readiness Assessment (SARA) 2017. 2017.

16. Government of Sierra Leone, Ministry of Health and Sanitation. NATIONAL HEALTH AND SANITATION POLICY 2021. 2021.

17. Government of Sierra Leone, Ministry of Health and Sanitation. NATIONAL HEALTH SECTOR STRATEGIC PLAN 2021-2025. 2021.

18. Govindaraj R, Herbst CH, Ajumobi O, Rockmore C, El Idrissi MDZE, Workie N, et al., editors. Strengthening Post-Ebola Health Systems: From Response to Resilience in Guinea, Liberia, and Sierra Leone. Washington, DC: World Bank; 2018.

19. Herrick C, Brooks A. The Binds of Global Health Partnership: Working out Working Together in Sierra Leone: Global Health Partnership. Medical Anthropology Quarterly. 2018 Dec;32(4):520–38.

20. Jalloh MF, Sengeh P, Ibrahim N, Kulkarni S, Sesay T, Eboh V, et al. Association of community engagement with vaccination confidence and uptake: A cross-sectional survey in Sierra Leone, 2019. J Glob Health. 2022 Feb 26;12:04006.

21. McPake B, Witter S, Ensor T, Fustukian S, Newlands D, Martineau T, et al. Removing financial barriers to access reproductive, maternal and newborn health services: The challenges and policy implications for human resources for health. Human Resources for Health. 2013 Sep 22;11(1).

22. Miller NP, Milsom P, Johnson G, Bedford J, Kapeu AS, Diallo AO, et al. Community health workers during the Ebola outbreak in Guinea, Liberia, and Sierra Leone. Journal of Global Health. 2018;8(2).

23. Narayan V, John-Stewart G, Gage G, O'Malley G. "If I had known, I would have applied": Poor communication, job dissatisfaction, and attrition of rural health workers in Sierra Leone. Human Resources for Health. 2018 Sep 24;16(1).

24. Nyhus HB, Kamara MM. Quality improvement in emergency service delivery: Assessment of knowledge and skills amongst emergency nurses at Connaught Hospital, Sierra Leone. African Journal of Emergency Medicine. 2017 Sep;7(3):113–7.

25. Oyerinde K, Harding Y, Amara P, Kanu R, Shoo R, Daoh K. The status of maternal and newborn care services in Sierra Leone 8 years after ceasefire. International Journal of Gynecology and Obstetrics. 2011;114(2):168–73.

26. Pieterse P, Lodge T. When free healthcare is not free. Corruption and mistrust in Sierra Leone's primary healthcare system immediately prior to the Ebola outbreak. International Health. 2015 Nov 1;7(6):400–4.

27. Squire JS, Hann K, Denisiuk O, Kamara M, Tamang D, Zachariah R. The Ebola outbreak and staffing in public health facilities in rural Sierra Leone: who is left to do the job? Public Health Action. 2017;7(1):47–54.

28. Squire JS, Hann K, Denisiuk O, Zachariah R. Staffing in public health facilities after the Ebola outbreak in rural Sierra Leone: How much has changed? F1000Research. 2020;8(793).

29. Tengbe SM, Kamara IF, Ali DB, Koroma FF, Sevalie S, Dean L, et al. Psychosocial impact of COVID-19 pandemic on front-line healthcare workers in Sierra Leone: an explorative qualitative study. BMJ Open. 2023 Aug;13(8):e068551.

30. Treacy L, Bolkan HA, Sagbakken M. Distance, accessibility and costs. Decision-making during childbirth in rural Sierra Leone: A qualitative study. Räisänen SH, editor. PLoS ONE. 2018 Feb 20;13(2):e0188280.

31. Vernooij E, Koker F, Street A. Responsibility, repair and care in Sierra Leone's health system. Social Science & Medicine. 2022 May;300:114260.

32. Willott C, Boyd N, Wurie H, Smalle I, Kamara TB, Davies JI, et al. Staff recognition and its importance for surgical service delivery: a qualitative study in Freetown, Sierra Leone. Health Policy and Planning. 2021 Mar 3;36(1):93–100.

33. Wilson S, Bah MM, George P, Caulker A, Holmer H, Leather AJM, et al. Challenges and solutions to providing surgery in Sierra Leone hospitals: A qualitative analysis of surgical provider perspectives. BMJ Open. 2022 Feb 1;12(2).

34. Witter S, Brikci N, Harris T, Williams R, Keen S, Jones A, et al. The Sierra Leone Free Health Care Initiative (FHCI): process and effectiveness review. HEART (Health and Education Advice and Resource Team); 2016.

35. Witter S, Wurie H, Bertone MP. The free health care initiative: How has it affected health workers in Sierra Leone? Health Policy and Planning. 2016 Feb 1;31(1):1–9.

36. Wurie H, Samai MH, Witter S. Staffing the public health sector in Sierra Leone, 2005-11: findings from routine data analysis'. Rebuild consortium. 2014;I(September):10–2.
